# Burnout Syndrome Among Critical Care Nurses After COVID-19 Pandemic: An International Single-Centre Study in Croatia and Poland

**DOI:** 10.3390/healthcare14091186

**Published:** 2026-04-28

**Authors:** Adriano Friganović, Biljana Filipović, Sabina Krupa-Nurcek, Kristian Civka, Cecilija Rotim, Jelena Slijepčević, Ana Brčina, Mohamed Mouhajir, Željko Vlaisavljević

**Affiliations:** 1 University Hospital Centre Zagreb, 10000 Zagreb, Croatia; adriano@hdmsarist.hr (A.F.); kristian.civka@hdmsarist.hr (K.C.); jelena.slijepcevic@kbc-zagreb.hr (J.S.); 2 Department of Nursing, University of Applied Health Sciences, 10000 Zagreb, Croatia; cecilija.rotim@rotim.hr; 3 Department of Nursing, Faculty of Health Studies, University of Rijeka, 51000 Rijeka, Croatia; 4 Department of Surgery, Institute of Medical Sciences, Medical College, University of Rzeszów, 35-310 Rzeszów, Poland; sabinakrupa@o2.pl; 5 Faculty of Dental Medicine and Health Osijek, Josip Juraj Strossmayer University of Osijek, 31000 Osijek, Croatia; 6 Rotim Polyclinic, 10000 Zagreb, Croatia; 7 NeuroSpine Hospital, 10000 Zagreb, Croatia; ana.brcina28996@gmail.com; 8 Department of Nursing, Mohammed V University, Rabat P.O. Box 554, Morocco; mouhajir10@gmail.com; 9 Department of Nursing, Medical College for Vocational Studies in Healthcare Medika, 11000 Belgrade, Serbia; kcszeljko@gmail.com

**Keywords:** burnout syndrome, critical care nurses, after COVID-19 period

## Abstract

**Background/Objectives:** Many frontline healthcare professionals had not previously faced a crisis of the magnitude of the COVID-19 pandemic, and prolonged exposure to high-stress clinical environments may adversely affect psychological well-being. This study aimed to assess and compare burnout severity among critical care nurses in two clinical settings—one hospital in Croatia and one in Poland—with particular attention to emotional exhaustion, depersonalization, and personal accomplishment in the post-pandemic period. **Methods:** A cross-sectional comparative design was conducted across two hospitals (Croatia and Poland). Data were collected from 346 critical care nurses between September and December 2023. Burnout was assessed using the Maslach Burnout Inventory Human Services Survey, analyzed primarily as continuous scores across its three dimensions. **Results:** No statistically significant differences were observed between the two groups in continuous burnout scores (Emotional Exhaustion *p* = 0.224, Depersonalization *p* = 0.852, Personal Accomplishment *p* = 0.636, total MBI score *p* = 0.394). Secondary cut-off-based analyses yielded some categorical differences, including a higher proportion classified as having high burnout in the Polish sample (43.2%) than in the Croatian sample (31.5%); however, these findings were exploratory and should not be interpreted as overriding the primary continuous-score results. Regression analyses demonstrated low explanatory power, with education level emerging as a significant predictor only in the Croatian sample (OR = 0.320, 95% CI: 0.125–0.824, *p* = 0.018). **Conclusions:** Burnout severity did not differ significantly between the two clinical settings when assessed using continuous measures. These findings suggest that burnout among ICU nurses may be driven primarily by shared occupational and organizational stressors rather than setting-specific differences. Categorical findings should be interpreted as complementary and exploratory.

## 1. Introduction

The World Health Organization (WHO) defines burnout as an “occupational phenomenon” and conceptualizes it as a result of chronic workplace stress that has not been successfully managed [1,2]. Burnout is typically associated with demanding work environments and suboptimal organizational and managerial practices, conditions that are frequently present in healthcare systems [1,3].

Burnout is particularly relevant in intensive care units (ICUs), where high-acuity clinical demands, time pressure, and staffing constraints often coincide with limited resources and organizational challenges, thereby increasing psychological strain and reducing job satisfaction [4]. Consequently, critical care nurses are consistently identified as a high-risk group for burnout due to their sustained exposure to critically ill patients, complex decision-making, and frequent emotional labor, often accompanied by insufficient institutional support [2,5,6].

The COVID-19 pandemic further highlighted the profound impact that large-scale health crises can have on healthcare systems and frontline staff, affecting multiple dimensions of professional and personal well-being [7]. Evidence suggests that ICU nurses experienced pronounced moral distress during the pandemic, driven by challenging care priorities, resource limitations, and high mortality rates, which have been associated with adverse health outcomes and workforce attrition in the post-pandemic period [8]. Moral distress, together with sustained physical and psychological fatigue, may further increase the risk of burnout in critical care settings [9].

Burnout is commonly assessed using validated psychometric instruments, most notably the Maslach Burnout Inventory (MBI), which conceptualizes burnout across three dimensions: Emotional Exhaustion (EE), Depersonalization (DP), and Personal Accomplishment (PA) [10]. EE reflects feelings of emotional depletion, DP refers to detached or cynical attitudes toward patients, and reduced PA indicates a diminished sense of professional efficacy and motivation [10]. These dimensions provide a structured framework for assessing burnout and comparing profiles across professional and organizational contexts.

Cross-cultural and cross-contextual research is important because burnout may manifest differently depending on healthcare system characteristics, staffing models, and institutional support mechanisms. Comparative studies can therefore contribute to the development of targeted, context-sensitive interventions aimed at improving working conditions and supporting workforce well-being. In Croatia and Poland, healthcare professionals faced different pandemic-related pressures, including variation in staffing adequacy, resource availability, and psychological support, which may have influenced burnout among ICU nurses in the post-COVID-19 period [11,12,13,14,15].

The healthcare systems of Croatia and Poland also differ in organizational structure and nursing roles, particularly with respect to professional autonomy, scope of practice, and workforce distribution. These differences may influence how occupational stress and burnout are experienced in clinical practice. For example, Poland has historically faced more pronounced nursing shortages and higher patient-to-nurse ratios, whereas Croatia operates within a smaller system with different staffing models and role expectations, providing a relevant basis for comparison at the level of clinical settings rather than national systems [11,12,13,14,15]. This context also underscores the importance of using instruments such as the MBI, which have been validated across diverse healthcare environments [5,6,10].

Beyond pandemic-related stressors, established contributors to burnout include inadequate staffing, unfavorable working conditions, strained interpersonal relationships, limited opportunities for professional development, and broader organizational constraints [16]. These factors have been associated with reduced job satisfaction, increased turnover intentions, and potentially compromised quality of care, highlighting burnout as both an occupational health and healthcare quality concern [17,18].

The aim of this study was to assess the prevalence and intensity of burnout among critical care nurses in two clinical settings—one hospital in Croatia and one hospital in Poland—in the post-COVID-19 period. The study also explored potential differences in burnout levels between nurses working in these two intensive care environments who were exposed to COVID-19 patients.

## 2. Materials and Methods

### 2.1. Design

This study employed a cross-sectional design to assess the prevalence and severity of burnout among critical care nurses in two clinical settings—one hospital in Croatia and one hospital in Poland—in the post-COVID-19 period. The study compared levels of Emotional Exhaustion, Depersonalization, and Personal Accomplishment between the two settings.

### 2.2. Study Setting

The study was conducted among nurses working in one hospital in Croatia and one hospital in Poland between September and December 2023.

### 2.3. Participants

A convenience sampling method was used. Questionnaires were distributed in paper form, and participants were informed that their responses would remain anonymous. The inclusion criteria were nurses who worked in an ICU during the COVID-19 pandemic and continued working in the ICU at the time of data collection. Participation was voluntary, and informed consent was obtained prior to inclusion in the study.

The final sample consisted of 346 nurses, including 163 from the hospital in Croatia and 183 from the hospital in Poland.

### 2.4. Instrument

Demographic data, including age, gender, education level, work experience, and workplace, were collected using a structured questionnaire. The primary research instrument was the Maslach Burnout Inventory (MBI) Human Services Survey, a validated translated version of the original instrument [19]. The MBI is an internationally validated tool that has previously been used in Croatia and widely applied in healthcare research.

The instrument consists of three subscales: Emotional Exhaustion (EE; 9 items), Depersonalization (DP; 5 items), and Personal Accomplishment (PA; 8 items).

Internal consistency reliability was high in both subsamples. For Emotional Exhaustion, Cronbach’s alpha was 0.90 in the Croatian subsample and 0.92 in the Polish subsample. For Depersonalization, Cronbach’s alpha was 0.75 in Croatia and 0.84 in Poland. For Personal Accomplishment, Cronbach’s alpha was 0.73 in Croatia and 0.75 in Poland. The overall MBI scale also demonstrated good internal consistency, with Cronbach’s alpha values of 0.76 in Croatia and 0.86 in Poland.

The sample structure is presented in Table 1. Participants were critical care nurses employed in ICUs in one hospital in Croatia and one hospital in Poland, comprising 47 male (13.6%) and 299 female (86.4%) nurses.

In both countries, most nurses had less than 5 years of ICU experience. In Croatia, participants displayed a broader range of experience, while in Poland, the only additional ICU experience category reported was 5–9 years. Additionally, the proportion of nurses with a master’s degree was significantly higher in Poland compared to Croatia (χ^2^ = 68.800, df = 2, *p* < 0.001), as was the proportion of female nurses (Fisher’s exact *p* < 0.001).

### 2.5. Data Collection

Prior to survey participation, the scope of the study was outlined in the questionnaire header, where respondents were also informed about the study’s ethical approval and consent process. The consent of the institutional ethics committee was documented, providing the committee’s reference number and contact details to the authors in case respondents wished to review the full consent documentation. Participants were notified that the study results would be used solely for research purposes. Data was collected in paper form and transferred to a data collection sheet.

### 2.6. Data Analysis

Data analysis was conducted using SPSS statistical software (IBM, Version 25.0) (IBM SPSS Statistics for Windows, Version 25.0. Armonk, NY, USA, IBM Corp.). The normality of distribution for continuous numerical variables was evaluated using the Shapiro–Wilk test. Descriptive indicators, including mean, standard deviation, median, and interquartile range, were presented since most variables deviated from a normal distribution. The internal consistency of scales was measured by Cronbach’s alpha coefficient.

For nominal (categorized) variables, both the frequency and percentage of respondents in each category were reported. To compare groups on categorical variables, the Chi-square test or Fisher’s exact test was applied as appropriate. Binary regression analysis was conducted to assess the influence of demographic variables on predicting high levels of burnout, defined by the cut-off value. Burnout was entered into the model as a binary criterion variable, with a score of 58 or higher on the MBI defining high burnout. This cut-off has been previously used in research by Friganović and Selič, and it is used to identify individuals with a high risk of burnout [20,21]. The significance level for Type I error was set at *p* = 0.05.

### 2.7. Ethical Considerations

This study was approved by the Ethical Committee of the University Hospital Centre Zagreb, Croatia, ethical approval number 8.1-23/138-2 (8 May 2023), and Medical Chamber in Gdansk, ethical approval number KB 6/24 (5 March 2023), Gdansk, Poland.

The research followed the ethical principles outlined in the Declaration of Helsinki [22]. To protect participants’ anonymity, no personal data that could lead to identification were collected. Participants had the right to withdraw from the study at any point without providing justification, and no identifying information was stored.

## 3. Results

### 3.1. Difference in Total MBI Scores

Due to statistically significant differences in the normality of distribution of the total MBI score and MBI subscales, the non-parametric Mann–Whitney test was used to compare the mean values of each subscale and the total MBI score between the countries. No statistically significant differences were found between the Croatian and Polish samples: Emotional Exhaustion (EE) *p* = 0.224, Depersonalization (DP) *p* = 0.852, Personal Accomplishment (PA) *p* = 0.636, and the overall MBI score *p* = 0.394. No statistically significant between-group differences were observed for any MBI subscale or for the total score. Additional descriptive analyses were then conducted.

### 3.2. Distribution of Burnout Questionnaire Subscales

The distribution of MBI subscale scores is presented according to manual-defined categories across all three dimensions. As a secondary exploratory analysis, MBI subscale scores were additionally categorized according to manually defined cut-off ranges (low, moderate, and high) to provide a descriptive overview of score distribution across the two samples. The percentages and numbers of respondents in each category are displayed in Table 2.

In this secondary descriptive analysis, the distribution of categorized subscale scores was broadly similar across the two samples for Emotional Exhaustion and Personal Accomplishment, while some variation was observed for Depersonalization.

Within the secondary categorical analysis, a difference was observed only for the distribution of Depersonalization categories (χ^2^ = 7.370, df = 2, *p* = 0.025). Given that no corresponding between-group difference was found in the primary continuous DP scores, this result should be interpreted cautiously as exploratory.

No statistically significant difference was observed between the two hospital samples in the proportion of nurses at high risk of burnout, as defined by the MBI Manual, which considers high composite scores in Emotional Exhaustion and Depersonalization coupled with a lower score in Personal Accomplishment. Specifically, 8.4% of nurses in Croatia and 12.6% in Poland fell into the high-risk category (Fisher’s exact *p* = 0.226).

### 3.3. Secondary Exploratory Categorization of Total Burnout Scores

As a secondary exploratory step, total MBI scores were additionally categorized using a previously applied cut-off for high burnout risk (score ≥ 58) [20,21]. This categorization was used to support descriptive interpretation and to define the binary outcome for regression analyses. These analyses were not intended to supersede the primary continuous-score comparisons.

Using this secondary cut-off-based categorization, 31.5% of nurses in Croatia and 43.2% in Poland were classified as having high burnout. Although this categorical comparison reached statistical significance (*p* = 0.028), it should be interpreted as exploratory and supportive only, because the primary continuous analyses did not demonstrate a statistically significant between-group difference in burnout severity (Table 3).

### 3.4. Logistic Regression Analysis of Burnout Predictors

Logistic regression analysis was conducted to determine whether specific demographic variables predicted a high degree of burnout, as defined by the cut-off value. Burnout was entered into the model as a binary criterion variable, with a score of 58 or higher on the MBI defining high burnout. Because the regression models used the secondary cut-off-based burnout classification as the binary outcome, separate binary logistic regression analyses were performed for each country, as shown in Table 4 and Table 5.

The predictors included in the models were age categories, gender, education level, and ICU work experience, with the last category of each variable serving as the reference.

The model explained 15% of the variance in high burnout scores (Nagelkerke R^2^ = 0.154). The only significant predictor of high burnout is the education level of Croatian ICU nurses. Nurses with a bachelor’s degree have a 68% lower likelihood of high burnout compared to those with a master’s degree (OR = 0.320, 95% CI: 0.125–0.824, *p* = 0.018).

The model explained only a small proportion of the variance in the outcome, specifically 4% (Nagelkerke R^2^ = 0.037), and none of the demographic variables included in the model emerged as significant predictors of high burnout in the Polish sample.

Given the low explanatory power of the demographic models, it is likely that unmeasured workplace and organizational factors are far more critical in determining burnout risk.

## 4. Discussion

The present study contributes to the post-pandemic burnout literature by showing that ICU nurses from the two clinical settings did not differ significantly in burnout severity when burnout was analyzed using continuous MBI scores. This is relevant because continuous measurement better captures the full distribution and intensity of emotional exhaustion, depersonalization, and personal accomplishment and therefore provides the most robust basis for between-group comparison. Conceptually, the findings support the interpretation of burnout as a response primarily shaped by chronic occupational and organizational stressors rather than by setting labels alone. In ICU settings, where workload intensity, emotional labor, staffing pressure, and exposure to critically ill patients are structurally embedded, this pattern is consistent with broader evidence emphasizing the importance of workplace well-being, coping, and organizational context in burnout development [21,23,24].

This interpretation is also compatible with previous findings in Croatian critical care nurses, which similarly highlighted burnout as a relevant occupational issue in intensive care settings [25]. More broadly, systematic evidence indicates that burnout in nursing staff is closely related to work engagement and to the quality of the working environment, reinforcing the view that organizational conditions may be more important than setting labels alone when interpreting between-group differences [26]. In this sense, the absence of continuous-score differences across the two settings appears theoretically plausible.

An important issue in interpreting the present findings is the divergence between the primary continuous analyses and the secondary categorical analyses. This pattern is methodologically and conceptually relevant. Continuous MBI scores retain gradations of severity across the full score range, whereas categorical cut-offs impose thresholds that can convert relatively modest shifts in score distribution into apparently clearer between-group contrasts. As a result, groups with similar average burnout severity may still differ in the proportion of participants falling above a predefined cut-off. This helps explain why the present study identified no statistically significant between-group differences in continuous burnout scores, while some differences emerged in the secondary cut-off-based analyses. This divergence does not necessarily indicate contradictory findings; rather, it reflects two different levels of interpretation—severity estimation versus threshold-based classification. In line with psychometric and methodological literature on burnout measurement, the continuous analyses are more appropriate for primary inferential comparison, whereas categorical findings are better understood as descriptive and hypothesis-generating [27,28,29,30,31].

The categorical difference observed for depersonalization may suggest some variation in how nurses in the two settings experience or express detached coping responses toward patients, but this interpretation remains tentative. Depersonalization is often discussed as a defensive adaptation to prolonged emotional overload and demanding care environments, while coping style may influence how burnout symptoms are expressed in practice [21,24]. However, because no corresponding difference was observed in the continuous DP scores, the present finding is better interpreted as a possible signal of threshold clustering within one part of the score distribution than as firm evidence of a substantively different depersonalization burden between the two groups.

The similarity in Personal Accomplishment across the two settings is also noteworthy. In the Maslach framework, this dimension reflects perceived competence, professional meaning, and effectiveness at work. Related literature suggests that burnout is closely linked to work engagement, perceived meaningfulness, and turnover intentions among nurses [21,27,28]. The absence of between-group differences in this domain may indicate that, despite differences in context, ICU nurses in both settings maintain a broadly comparable sense of professional efficacy. At the same time, this result may reflect a paradox frequently described in burnout research: professionals may continue to perceive their work as meaningful even when operating under sustained strain and emotional exhaustion. This interpretation is also broadly consistent with wider burnout literature in nursing populations [29].

The regression analyses further support the interpretation that burnout in this study was only weakly explained by individual demographic variables. Although education level emerged as a significant predictor in the Croatian sample, the low Nagelkerke R^2^ values in both models indicate limited explanatory capacity overall. This suggests that burnout risk in ICU nurses is unlikely to be adequately understood through demographic profiling alone. Rather, the findings are more consistent with approaches that place greater emphasis on workplace and organizational determinants, including support structures, coping resources, and the psychosocial climate of care [23,24,26]. In this sense, the regression results do not merely show a lack of demographic effects; they indirectly reinforce the broader interpretation that workplace context, rather than individual background characteristics, is likely to be the more important level of explanation.

The present findings underscore the importance of analytical alignment between measurement strategy and interpretive claims. When the aim is to compare burnout severity between groups, continuous scores offer a stronger inferential basis because they preserve information and reduce the risk of threshold-driven distortion. Categorical classifications remain useful for descriptive purposes, prevalence-oriented reporting, and comparison with prior studies, but they should not be granted equal interpretive status when primary continuous comparisons are null.

### Study Limitations

This study included ICU nurses from one hospital in Croatia and one hospital in Poland, which limits the generalizability of the findings to other healthcare professions, institutions, or clinical settings. Accordingly, the results should be interpreted as reflecting two specific clinical environments rather than broader national contexts. Future research should include larger and more diverse samples across multiple institutions and healthcare systems to provide a more comprehensive understanding of burnout and its determinants.

Another important limitation is the lack of data on key organizational and workplace factors that are likely to influence burnout. Specifically, no information was collected on staffing ratios, workload, availability of personal protective equipment (PPE), access to psychological support services, or pandemic-specific institutional policies. In the absence of these variables, interpretations related to organizational conditions and workplace stressors remain indirect and should therefore be interpreted with caution. Future studies should incorporate these factors to enable a more robust analysis of the determinants of burnout in critical care settings.

## 5. Conclusions

In conclusion, the primary analyses of continuous MBI scores showed no statistically significant differences in burnout severity between ICU nurses in the Croatian and Polish clinical settings. Secondary categorical analyses identified some cut-off-based variation; however, these findings were exploratory and supportive only and should not be interpreted as superseding the primary continuous-score results.

Overall, the findings emphasize the importance of addressing shared occupational and organizational stressors when developing strategies to reduce burnout among ICU nurses, rather than focusing on broader contextual or national differences.

## Figures and Tables

**Table 1:** Number and percentage of respondents according to collected demographic data.

		Country				Total	
		Croatia		Poland		n	%
		n	%	n	%		
Gender	Male	40	24.5	7	3.8	47	13.6
	Female	123	75.5	176	96.2	299	86.4
Total		163	100	183	100	346	100
Age categories	18–25 yrs	39	24.1	64	35	103	29.9
	26–35 yrs	82	50.6	90	49.2	172	49.9
	36–45 yrs	29	17.9	16	8.7	45	13
	46–55 yrs	11	6.8	13	7.1	24	7
	>55 yrs	1	0.6			1	0.3
Education	vocational	39	23.8	3	1.6	42	12.1
	bachelor	89	54.3	71	38.8	160	46.1
	master	36	22	109	59.6	145	41.8
Working experience in ICU	≤5 yrs	82	50.9	100	54.6	182	52.9
	5–9 yrs	45	28	83	45.4	128	37.2
	10–14 yrs	12	7.5			12	3.5
	15–20 yrs	5	3.1			5	1.5
	≥20 yrs	17	10.6			17	4.9
Workplace/division	Cardiac surgery	22	13.5	44	24	66	19.1
	neurosurgery	12	7.4			12	3.5
	pediatric neonatal	18	11			18	5.2
	medical	22	13.5			22	6.4
	general surgery	30	18.4	37	20.2	67	19.4
	coronary	13	8			13	3.8
	neurology	8	4.9	41	22.4	49	14.2
	other	38	23.3	61	33.3	99	28.6

**Table 2:** Percentages and numbers of respondents according to levels of Emotional Exhaustion (EE), Personal Accomplishment (PA), and Depersonalization (DP) in both countries.

		Country				Total		*p*
		Croatia		Poland		n	%	
		n	%	n	%			
EE	low	33	19.5	40	21.9	73	20.7	0.416
	moderate	63	37.3	56	30.6	119	33.8	
	high	73	43.2	87	47.5	160	45.5	
PA	low	45	26.6	48	26.2	93	26.4	0.991
	moderate	58	34.3	64	35	122	34.7	
	high	66	39.1	71	38.8	137	38.9	
DP	low	95	55.9	96	52.5	191	54.1	0.025
	moderate	50	29.4	40	21.9	90	25.5	
	high	25	14.7	47	25.7	72	20.4	

**Table 3:** Proportion of respondents classified as having high burnout according to the total MBI score.

		Country				Total		*p*
		Croatia		Poland		n	%	
		n	%	n	%			
MBI in 2 categories	Low/medium burnout	115	68.5	104	56.8	219	62.4	0.028
	high burnout	53	31.5	79	43.2	132	37.6	

**Table 4:** Results of binary regression analysis for burnout in Croatia.

	B	df	Sig.	OR	95% CI	
Age categories		3	0.120			
Age categories (18–25 yrs)	1.772	1	0.162	5.882	0.491	−70.514
Age categories (26–35 yrs)	1.924	1	0.103	6.852	0.677	−69.349
Age categories (36–45 yrs)	0.214	1	0.841	1.239	0.154	−9.974
Gender (Male)	0.412	1	0.346	1.510	0.641	−3.554
Education		2	0.019			
Education (vocational)	−0.088	1	0.874	0.916	0.309	−2.715
Education (bachelor)	−1.138	1	0.018	0.320	0.125	−0.824
Working experience in ICU (<5 yrs)		4	0.135			
Working experience in ICU (5–9 yrs)	−0.687	1	0.480	0.503	0.075	−3.386
Working experience in ICU (10–14 yrs)	−1.537	1	0.194	0.215	0.021	−2.186
Working experience in ICU (15–20 yrs)	0.282	1	0.818	1.326	0.120	−14.649
Constant	0.570	1	0.537	0.567		

**Table 5:** Results of binary regression analysis for burnout in Poland.

	B	df	Sig.	OR	95% CI	
Age categories		3	0.210			
Age categories (18–25 yrs)	−1.202	1	0.061	0.301	0.085	−1.059
Age categories (26–35 yrs)	−0.700	1	0.258	0.497	0.148	−1.670
Age categories (36–45 yrs)	−0.588	1	0.448	0.555	0.122	−2.535
Gender (Male)	0.244	1	0.762	1.276	0.264	−6.174
Education		2	0.703			
Education (vocational)	−0.855	1	0.503	0.425	0.035	−5.187
Education (bachelor)	−0.186	1	0.565	0.830	0.440	−1.567
Working experience in ICU (<5 yrs)	0.000	1	0.999	1.000	0.550	−1.819
Constant	0.611	1	0.320	1.842		

## Data Availability

Study data are available upon request from the corresponding author.

## Abbreviations

The following abbreviations are used in this manuscript:

BS	Burnout Syndrome
COVID-19	Coronavirus Disease 2019
DP	Depersonalization
EE	Emotional Exhaustion
ICU	Intensive Care Unit
MBI	Maslach Burnout Inventory
PA	Personal Accomplishment
WHO	World Health Organization

## References

[1] World Health Organization (2019). Burn-Out an “Occupational Phenomenon”: International Classification of Diseases.

[2] De Hert S. (2020). Burnout in healthcare workers: Prevalence, impact and preventative strategies. Local Reg. Anesth..

[3] de Beer L.T., Schaufeli W.B., De Witte H., Hakanen J.J., Shimazu A., Glaser J., Seubert C., Bosak J., Sinval J., Rudnev M. (2020). Measurement invariance of the burnout assessment tool (BAT) across seven cross-national representative samples. Int. J. Environ. Res. Public Health.

[4] Quesada-Puga C., Izquierdo-Espin F.J., Membrive-Jiménez M.J., Aguayo-Estremera R., La Fuente G.A.C.-D., Romero-Béjar J.L., Gómez-Urquiza J.L. (2024). Job satisfaction and burnout syndrome among intensive-care unit nurses: A systematic review and meta-analysis. Intensiv. Crit. Care Nurs..

[5] Baugh J.J., Takayesu J.K., White B.A., Raja A.S. (2020). Beyond the Maslach burnout inventory: Addressing emergency medicine burnout with Maslach’s full theory. J. Am. Coll. Emerg. Physicians Open..

[6] Carvalho de Sousa C. (2024). Combined effects of gender, race, and occupational stressors on mental health. Rev. Bras. Saude Ocup..

[7] de Vries N., Maniscalco L., Matranga D., Bouman J., de Winter J.P. (2024). Determinants of intention to leave among nurses and physicians in a hospital setting during the COVID-19 pandemic: A systematic review and meta-analysis. PLoS ONE.

[8] Gehrke P., Campbell K., Tsang J.L.Y., Hannon R.A., Jack S.M. (2024). Canadian intensive care unit nurses’ responses to moral distress during the COVID-19 pandemic, and their recommendations for mitigative interventions. J. Adv. Nurs..

[9] Soares J.P., Lopes R.H., Mendonça P.B.S., Silva C.R.D.V., Rodrigues C.C.F.M., Castro J.L. (2023). Use of the Maslach Burnout Inventory among public health care professionals: Scoping review. JMIR Ment. Health.

[10] Ajhenberger S., Hodak J., Vadlja I., Anić D. (2020). Job satisfaction—A predictor of working efficiency and intentions to remain in nursing. Croat. Nurs. J..

[11] Izdebski Z., Kozakiewicz A., Białorudzki M., Dec-Pietrowska J., Mazur J. (2023). Occupational burnout in healthcare workers, stress and other symptoms of work overload during the COVID-19 pandemic in Poland. Int. J. Environ. Res. Public Health.

[12] Salopek-Žiha D., Hlavati M., Gvozdanović Z., Gašić M., Placento H., Jakić H., Klapan D., Šimić H. (2020). Differences in distress and coping with the COVID-19 stressor in nurses and physicians. Psychiatr. Danub..

[13] Szepietowski J.C., Krajewski P., Biłynicki-Birula R., Poznański P., Krajewska M., Rymaszewska J., Matusiak Ł. (2020). Mental health status of health care workers during the COVID-19 outbreak in Poland: One region, two different settings. Dermatol. Ther..

[14] Szwamel K., Kaczorowska A., Lepsy E., Mroczek A., Golachowska M., Mazur E., Panczyk M. (2022). Predictors of the occupational burnout of healthcare workers in Poland during the COVID-19 pandemic: A cross-sectional study. Int. J. Environ. Res. Public Health.

[15] Vlah Tomičević S., Bralić Lang V. (2021). Psychological outcomes amongst family medicine healthcare professionals during COVID-19 outbreak: A cross-sectional study in Croatia. Eur. J. Gen. Pract..

[16] Maslach C., Jackson S.E. (1981). The measurement of experienced burnout. J. Occup. Behav..

[17] Delobelle P., Rawlinson J.L., Ntuli S., Malatsi I., Decock R., Depoorter A.M. (2011). Job satisfaction and turnover intent of primary healthcare nurses in rural South Africa: A questionnaire survey. J. Adv. Nurs..

[18] Efobi O.C. (2022). Poor management skills: A contributing factor to high turnover rate in nursing homes. Fortune J. Health Sci..

[19] Maslach C. (1979). Burned-out. Can. J. Psychiatr. Nurs..

[20] Friganović A., Selič P. (2020). Levels of burnout syndrome in Croatian critical care nurses: A cross-sectional study. Psychiatr. Danub..

[21] Maslach C., Leiter M.P. (2016). Understanding the burnout experience: Recent research and its implications for psychiatry. World Psychiatry.

[22] World Medical Association (2024). WMA Declaration of Helsinki—Ethical Principles for Medical Research Involving Human Subjects.

[23] Makina-Zimalirana N., Bisnauth M., Shangase N., Davies N., Jiyane A., Buthelezi F., Rees K. (2023). Workplace wellbeing among health care workers providing HIV services in primary care in Johannesburg: A mixed methods study. Front. Public Health.

[24] Maresca G., Corallo F., Catanese G., Formica C., Lo Buono V. (2022). Coping strategies of healthcare professionals with burnout syndrome: A systematic review. Medicina.

[25] Friganović A., Selič P. (2021). Where to Look for a Remedy? Burnout Syndrome and its Associations with Coping and Job Satisfaction in Critical Care Nurses-A Cross-Sectional Study. Int. J. Environ. Res. Public Health.

[26] Vargas-Benítez M.Á., Izquierdo-Espín F.J., Castro-Martínez N., Gómez-Urquiza J.L., Albendín-García L., Velando-Soriano A., Cañadas-De la Fuente G.A. (2023). Burnout syndrome and work engagement in nursing staff: A systematic review and meta-analysis. Front. Med..

[27] Mohamed S.A., Hendy A., Ezzat Mahmoud O., Mohamed Mohamed S. (2022). Mattering perception, work engagement and its relation to burnout amongst nurses during coronavirus outbreak. Nurs. Open..

[28] Karimi L., Raei M., Parandeh A. (2022). Association between dimensions of professional burnout and turnover intention among nurses working in hospitals during coronavirus disease (COVID-19) pandemic in Iran based on structural model. Front. Public Health.

[29] Gómez-Urquiza J.L., Velando-Soriano A., Martos-Cabrera M.B., Cañadas G.R., Albendín-García L., la Fuente G.A.C.-D., Aguayo-Estremera R. (2023). Evolution and treatment of academic burnout in nursing students: A systematic review. Healthcare.

[30] Lin C.Y., Alimoradi Z., Griffiths M.D., Pakpour A.H. (2022). Psychometric properties of the Maslach Burnout Inventory for Medical Personnel (MBI-HSS-MP). Heliyon.

[31] Forné C., Yuguero O. (2022). Factor structure of the Maslach Burnout Inventory Human Services Survey in Spanish urgency healthcare personnel: A cross-sectional study. BMC Med. Educ..

